# The opioid peptide dynorphin A induces leukocyte responses via integrin Mac-1 (α_M_β_2_, CD11b/CD18)

**DOI:** 10.1186/s12990-015-0027-0

**Published:** 2015-06-03

**Authors:** Nataly P. Podolnikova, Julie A. Brothwell, Tatiana P. Ugarova

**Affiliations:** From the Center for Metabolic and Vascular Biology, School of Life Sciences, Arizona State University, Tempe, AZ 85287 USA

**Keywords:** Dynorphin A, Integrin Mac-1 (CD11b/CD18), Leukocytes, Opioid peptides, Adhesion molecules

## Abstract

**Background:**

Opioid peptides, including dynorphin A, besides their analgesic action in the nervous system, exert a broad spectrum of effects on cells of the immune system, including leukocyte migration, degranulation and cytokine production. The mechanisms whereby opioid peptides induce leukocyte responses are poorly understood. The integrin Mac-1 (α_M_β_2_, CD11b/CD18) is a multiligand receptor which mediates numerous reactions of neutrophils and monocyte/macrophages during the immune-inflammatory response. Our recent elucidation of the ligand recognition specificity of Mac-1 suggested that dynorphin A and dynorphin B contain Mac-1 recognition motifs and can potentially interact with this receptor.

**Results:**

In this study, we have synthesized the peptide library spanning the sequence of dynorphin AB, containing dynorphin A and B, and showed that the peptides bound recombinant α_M_I-domain, the ligand binding region of Mac-1. In addition, immobilized dynorphins A and B supported adhesion of the Mac-1-expressing cells. In binding to dynorphins A and B, Mac-1 cooperated with cell surface proteoglycans since both anti-Mac-1 function-blocking reagents and heparin were required to block adhesion. Further focusing on dynorphin A, we showed that its interaction with the α_M_I-domain was activation independent as both the α7 helix-truncated (active conformation) and helix-extended (nonactive conformation) α_M_I-domains efficiently bound dynorphin A. Dynorphin A induced a potent migratory response of Mac-1-expressing, but not Mac-1-deficient leukocytes, and enhanced Mac-1-mediated phagocytosis of latex beads by murine IC-21 macrophages.

**Conclusions:**

Together, the results identify dynorphins A and B as novel ligands for Mac-1 and suggest a role for the Dynorphin A-Mac-1 interactions in the induction of nonopiod receptor-dependent effects in leukocytes.

**Electronic supplementary material:**

The online version of this article (doi:10.1186/s12990-015-0027-0) contains supplementary material, which is available to authorized users.

## Background

Dynorphins are endogeneous opioid peptides released from injured neurons. By binding to the G-protein-coupled κ-opioid receptors found throughout the central and peripheral nervous systems they exert protective analgesic effects. At pathophysiological conditions, however, in which the dynorphin concentrations are elevated, these peptides may induce proalgesic effects and contribute to neurodegeneration largely acting at glutamate receptors (Reviewed in [1]). In addition to neurons, dynorphins are synthesized and released by stimulated leukocytes, including resident immune cells and cells that migrate to inflamed tissues from circulation (Reviewed in [2–4]). Since tissue injury is associated with an inflammatory reaction and pain, secretion of leukocyte-derived opioid peptides, including dynorphins, and their interaction with opioid receptors on peripheral sensory neurons presents an important mechanism that contributes to counteracting inflammatory pain [4].

Dynorphin A (Dyn A) is one of several processed neuropeptides (collectively referred to as “dynorphins”) derived from the prodynorphin protein. Although the mechanism for prodynorphin processing in leukocytes has not been fully elucidated, in neurons the proprotein is cleaved by convertases, primarily PC2, resulting in the generation of dynorphin AB (Dyn AB; also known as “big dynorphin”) consisting of Dyn A (residues 1–17) and dynorphin B (Dyn B; residues 20–32). Dyn A can be cleaved further, giving rise to shorter peptides, for example Dyn A (1–8).

Apart from their effects on κ-opioid receptors on neurons, Dyn A exerts numerous effects on leukocytes, particularly myeloid cells. Dyn A has been shown to increase secretion of IL-1 and TNFα by macrophages [5, 6], enhance phagocytosis by mouse peritoneal macrophages [7], stimulate macrophage superoxide generation [8], enhance macrophage zymosan-triggered oxidative burst [9], decrease nitric oxide release induced by LPS and IFN-γ [10] and induce degranulation and histamine release from rat mast cells [11–13]. In addition, Dyn A (residues 1–13) and Dyn A (residues 1–9) stimulate human mononuclear cell and neutrophil chemotaxis [14, 15]. Opioid receptors have been demonstrated in immune cells (Reviewed in [4]). However, even though some of the observed effects of Dyn A are mediated by opioid receptors, such responses as phagocytosis, degranulation and chemotaxis could not be blocked or were blocked partially by naloxone, the classical opioid receptor antagonist [7, 11, 15]. These results imply that both opioid and nonopiod receptors may be involved.

The integrin α_M_β_2_ (Mac-1, CD11b/CD18) is the major receptor expressed on the surface of phagocytic leukocytes, which mediates numerous cellular responses of these cells during the immune-inflammatory response. Ligand engagement by Mac-1 initiates phagocytosis, migration, degranulation, and expression of cytokines and other bioactive molecules [16]. A remarkable feature of Mac-1 is its ability to bind a multitude of diverse ligands. We have recently determined a unifying principle that governs the broad ligand binding specificity exhibited by Mac-1. We showed that the α_M_I-domain, a ligand binding region of Mac-1, recognizes short sequences containing positively charged and hydrophobic residues that are present in many Mac-1 ligands. The Mac-1 recognition sequences are exemplified by HyBHy, HyHyBHy, HyBHyHy and HyHyBHyHy motifs, where Hy represents any hydrophobic residue and B is basic residues with a preference for Arg [17]. Furthermore, negatively charged and polar residues are depleted in the α_M_I-domain binders. We further proposed that cationic proteins and peptides, which are stored in myeloid cells and remarkably enriched in such motifs, represent a group of Mac-1 ligands. These proteins/peptides, when released from activated or damaged cells, would alarm the immune system by triggering responses by binding to Mac-1. The sequence analyses of Dyn A (YGGFLRRIRPKLKWDNQ) and Dyn B (YGGFLRRQFKVVT) have indicated that these peptides contain several Mac-1 binding sites (underlined), and thus, may be the Mac-1 candidate ligands.

In the present study we have examined the interactions of Dyn A and Dyn B with recombinant α_M_I-domain and Mac-1-expressing cells and showed that these opioid peptides are Mac-1 ligands. Further focusing on Dyn A, we demonstrate that its binding to Mac-1 mediates directed cell migration of U937 monocytic cells and murine macrophages, and enhances phagocytosis by murine macrophages. Our results imply that binding of Dyn A to Mac-1 may account for some nonopioid effects of this peptide on leukocytes. They also suggest that Dyn A may perform dual functions during neuroinflammation, i.e., produce analgesia acting via its cognate opioid receptors on neurons and also alarm the immune cells, causing them to migrate to sites of tissue injury and activate other leukocyte responses.

## Results

### Screening of the peptide library spanning the sequence of dynorphin AB for α_M_I-domain binding

**Fig. 1:**
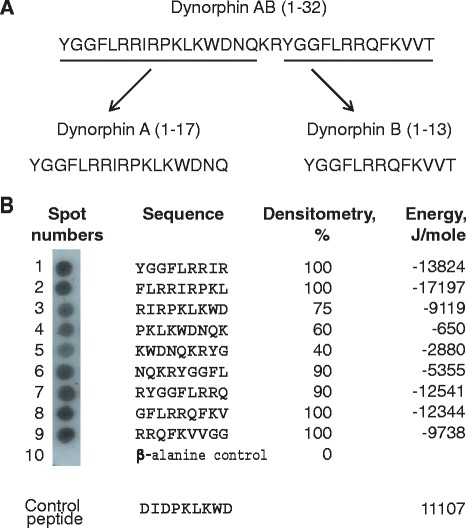
Screening of the peptide library spanning the sequence of dynorphin AB for α_M_I-domain binding. **a**, Amino acid sequence of dynorphin AB (residues 1–32). **b**, Cellulose membrane with the assembled peptide library consisting of 9-mer peptides with three-residue offset was incubated with ^125^I-labeled α_M_I-domain and subjected to autoradiography followed by densitometry. The α_M_I-domain binding was observed as dark spots. Spot #10 contains only the β-Ala spacer. The α_M_I-domain binding was expressed as a percentage of binding to peptide #1, which was assigned a value 100 % and is shown as the numbers on the right of each sequence. The peptide energies that serve as a measure of probability each peptide can interact with the α_M_I-domain were calculated as described [17]

To test the hypothesis that leukocyte integrin Mac-1 binds opioid peptides, we screened the peptide library spanning the sequence of dynorphin AB (Fig. 1a) for α_M_I-domain binding. The library, consisting of 9-mer peptides with a 3-residue offset (Fig. 1b) covalently attached to a cellulose membrane, was incubated with the ^125^I-labeled α_M_I-domain after which the α_M_I-domain binding was visualized by autoradiography and analyzed by densitometry. The densitometry results demonstrated that all peptides interacted with the α_M_I-domain, albeit to somewhat different extents (Fig. 1b). A control spot containing only the β-alanine spacer (spot 10) was negative. The dynorphin-derived peptides were also analyzed by the computer program IRMA, which determines the capacity of each peptide in the peptide libraries to interact with the α_M_I-domain [17]. The program assigns each peptide the energy value which serves as a measure of probability that the α_M_I-domain binds this sequence: the lower the energy, the higher the likelihood that the sequence binds the α_M_I-domain. As previously determined, strong α_M_I-domain binding peptides derived from various Mac-1 ligands have energy values in the range of −20 to 6 kJ/mole. The analyses showed a good relationship between the energy scores and the α_M_I-binding activity of dynorphin AB-derived peptides revealed in peptide scans (Fig. 1b). In line with previous findings [17, 18], peptides enriched in positively charged and hydrophobic residues had the highest affinities for the α_M_I-domain (spots 1, 2 and 6 through 10). In particular, the YGGFLRRIR and FLRRIRPKL peptides (spots 1 and 2), contain the strong overlapping α_M_I-domain binding clusters FLRRI and IRP, which conform to the α_M_I-domain binding motif HyHyBHy and HyBHy. The presence of negatively charged residues in peptides present in spots 3, 4 and 5 weakened their α_M_I-domain binding activity.

To confirm that α_M_I-domain binding to dynorphin AB-derived peptides depends on basic residues and thus to further substantiate the specificity of the interaction, we synthesized substitutional peptide libraries in which Lys and Arg were changed to Asp and Glu, respectively (Additional file 1: Figure S1). The results demonstrated that mutations of a single basic residue resulted in a decrease of peptide’s ability to bind the α_M_I domain and double or triple mutations within 9-mer peptides abolished *α*_M_I domain binding. Together, these results indicate that two non-overlapping sections of dynorphin AB, encompassing the sequences of Dyn A and Dyn B, contain the *α*_M_I-domain binding sites.

### Binding of the α_M_ I-domain to dynorphin A is activation-independent

**Fig. 2:**
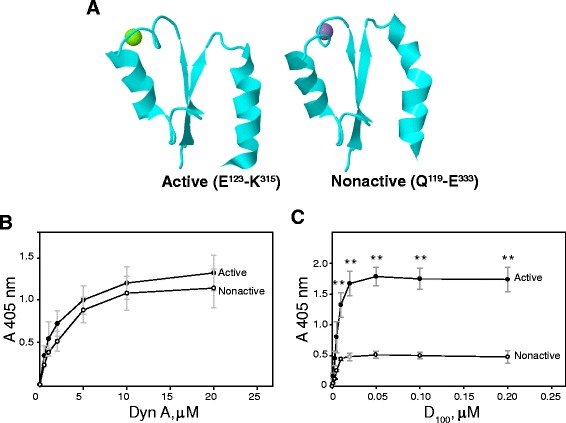
Binding of the active and nonactive α_M_I-domain conformers to dynorphin. **a**, the 3D structure of active (PDB ID 1ido) and nonactive (PDB ID 1jlm) α_M_I-domains are shown as ribbon diagrams. The wells of microtiter plates were coated with different concentrations of dynorphin A (**b**) and the D_100_ fragment of fibrinogen (**c**). The active (●) and nonactive (○) recombinant α_M_I-domains containing GST fusion parts were added to the wells and incubated for 1.5 h at 22 °C. Bound α_M_I-domains were detected with an anti-GST mAb followed by an AP-conjugated secondary antibody. Data are the means of triplicate determinations, and error bars represent S.E. Representative experiments in **b** (n = 4) and **c** (n = 4) are shown. For multiple comparisons, individual concentrations were tested using *t*-test with p-value of 0.05 corrected by Bonferroni method and accepting values of 0.007 as significant (**). No significant difference in binding of active and nonactive α_M_I-domains to Dyn A was found

Previous studies demonstrated that the *α*_M_I-domain exists in two different conformations, active and nonactive, with the length of the C-terminal α7 helix regulating its activation state [19]. Screening of the peptide libraries in the experiments above was performed using the active form of the α_M_I-domain, which encompasses residues α_M_Glu^123^-Lys^315^. We produced the active and nonactive forms of the α_M_I-domain (α_M_Gln^119^-Glu^333^) (Fig. 2a) as fusions with GST and compared their binding to immobilized Dyn A using solid-phage binding assays. As shown in Fig. 2b, there was no significant difference between the two α_M_I-domains. As a control, binding of GST to Dyn A was less than 5 % of the α_M_I-domains (not shown). As another essential control, the binding of active α_M_I-domain to the fibrinogen D_100_ fragment, the activation-dependent Mac-1 ligand [18], was ~3.5-fold higher than that of the nonactive form (Fig. 2c). These results indicate that the α_M_I-domain binding to Dyn A is not activation dependent.

### Dynorphins A and B support cell adhesion

**Fig. 3:**
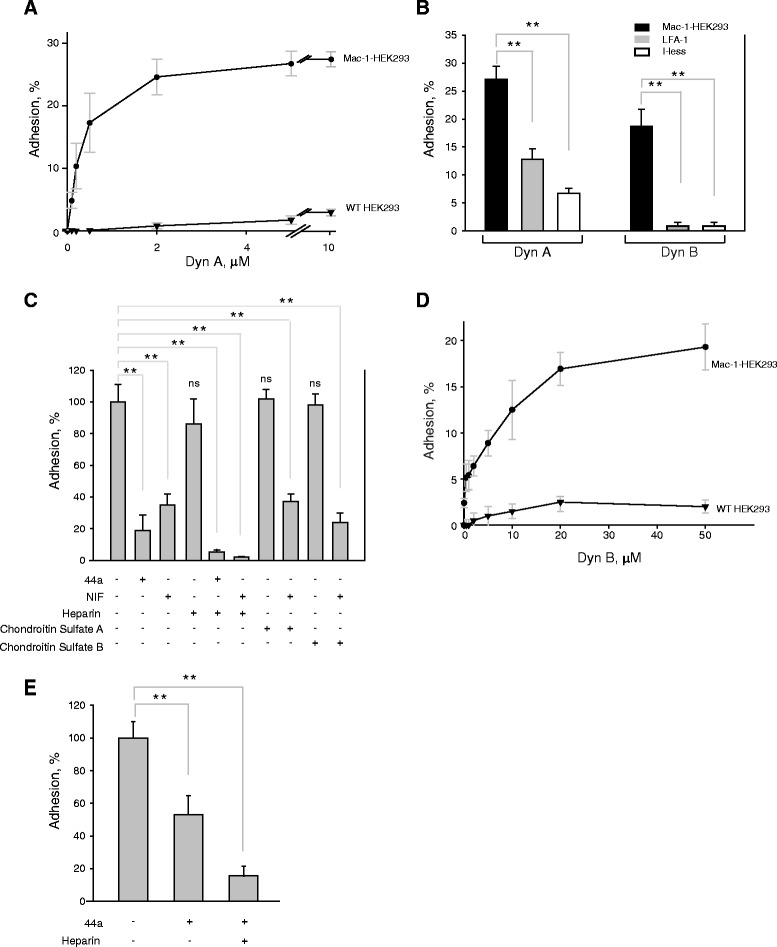
Adhesion of Mac-1-expressing cells to Dyn A and Dyn B. **a**, Mac-1-expressing and wild-type HEK293 cells were labeled with calcein and 5x10^4^ cells were added to the wells coated with different concentrations of the Dyn A. After 30 min at 37 °C, nonadherent cells were removed and fluorescence was measured. The data shown are means of triplicate determinations, and error bars represent S.E. The figure is representative of 8 experiments. **b** *,* adhesion of HEK293 cells expressing Mac-1, LFA and the I-less form of Mac-1 to Dyn A and Dyn B immobilized at 10 μM was determined as described in *A*. **c**, calcein-labeled Mac-1-HEK293 cells were preincubated without or with 10 μg/ml of anti-α_M_ mAb 44a or 1 μg/ml NIF for 15 minutes. For testing the role of glucosaminoglycans in cell adhesion, dynorphin A-coated wells were preincubated with 100 μl of 10 μg/ml of chondroitin sulfate A, chondroitin sulfate B and heparin. Aliquots (5x10^4^cells) were added to the wells coated with 2 μM dynorphin A and adhesion was performed as described in *A*. Adhesion in the absence of Mac-1 inhibitors and glucosaminoglycans was assigned a value of 100 %. The data shown are the means ± S.E. from four separate experiments each with triplicate measurements. **d** *,* microtiter plate wells were coated with different concentrations of dynorphin B and adhesion of Mac-1-expressing and wild-type HEK293 cells was determined as described in *A*. The data shown are means of triplicate determinations, and error bars represent S.E. The figure is representative of 5 experiments. **e**. calcein-labeled Mac-1-HEK293 cells were preincubated without or with 10 μg/ml of mAb 44a and heparin, added to the wells coated with 20 μM of dynorphin B and adhesion was performed as described in *A.* Adhesion in the absence of inhibitors was assigned a value of 100 %. The data shown are the means ± S.E from three separate experiments each with triplicate measurements. **p < 0.01; ns, no significant difference

The ability of the α_M_I-domain to bind Dyn A and Dyn B suggests that these peptides can interact with the whole receptor expressed on cells. A standard approach to test the interaction between Mac-1 and their ligands is an adhesion assay in which HEK293 cells stably transfected with Mac-1 are examined for their ability to bind the immobilized ligands [20, 18]. As shown in Fig. 3a, Mac-1-HEK293 cells strongly adhered to wells coated with Dyn A. Cell adhesion was dose-dependent with saturable adhesion achieved with the coating concentration of peptide at ~2 μM. By contrast, wild-type HEK293 cells adhered only slightly to Dyn A, indicating that β_1_ integrins, the major group of integrins on these cells, minimally contribute to adhesion. As additional controls, HEK293 cells expressing the homologous β_2_ integrin LFA-1 (α_L_β_2_, CD11a/CD18) or the I-less form of Mac-1 adhered to Dyn A less efficiently (13 ± 2 % and 7 ± 1 %, respectively) (Fig. 3b). Control peptide DIDPKLKWD also failed to support adhesion of Mac-1-HEK293 cells (not shown). To further establish the specificity of Mac-1 binding to Dyn A, several anti-Mac-1 reagents were tested for their ability to inhibit adhesion. Function-blocking mAb 44a directed against the α_M_ integrin subunit and the specific inhibitor of Mac-1, NIF, inhibited adhesion of Mac-1-HEK293 cells (Fig. 3c). At 2 μM of Dyn A, mAb 44a and NIF inhibited adhesion by 81 ± 8 % and 65 ± 7 %, respectively. However, inhibition was not complete, suggesting that other cell structures are involved in binding.

We previously demonstrated that soluble heparin inhibits cell adhesion to several Mac-1 peptide ligands, indicating that cell surface heparan sulfate proteoglycans (HSPGs) cooperate with Mac-1 [21, 22]. Since Dyn A is a highly positively charged molecule and is expected to interact with negatively charged HSPGs, we examined the involvement of HSPGs in adhesion of Mac-1-expressing HEK293 cells to Dyn A. The wells, coated with 2 μM Dyn A, were incubated with heparin for 20 min and cells were added. As shown in Fig. 3c, heparin at 10 μg/ml exhibited slight inhibition, but the combination of heparin with either mAb 44a or NIF produced almost complete inhibition (≥95 %) suggesting that cell surface HSPGs are involved in Mac-1-mediated adhesion to Dyn A. Two other less negatively charged glycosaminoglycans, chondroitin sulfate A and B, were tested alone or in combination with NIF and found to not exert a statistically significant inhibitory effect (Fig. 3c).

Figure 3d shows that Mac-1-HEK293, but not wild-type HEK293 cells, adhered to immobilized Dyn B; however, on a molar basis, higher coating concentrations of the peptide compared to Dyn A were required for cell adhesion to occur. While ~0.2-0.5 μM of Dyn A was required to reach 50 % of maximal cell adhesion, the same extent of adhesion occurred at ~7.5 μM of Dyn B. The HEK293 cells expressing integrin LFA-1 and the I-less form of Mac-1 failed to adhere to Dyn B (Fig. 3b). Similar to Dyn A, cell adhesion was partially inhibited by anti-α_M_ mAb 44a (52 ± 4 %) and effectively blocked by 84 ± 1 % in the presence of mAb 44a and heparin (Fig. 3d). Taken together, these observations indicate that both Dyn A and Dyn B fulfill the specification of typical Mac-1 ligands.

### Soluble dynorphins inhibit cell adhesion to the Mac-1 ligand fibrinogen

**Fig. 4:**
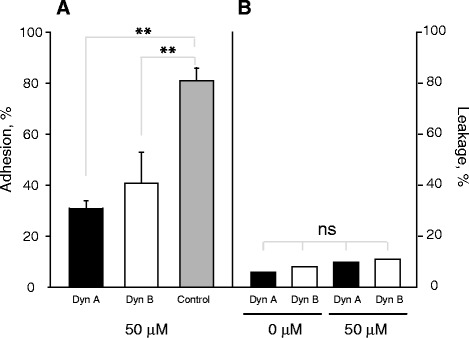
Effect of soluble Dyn A on Mac-1-mediated cell adhesion to fibrinogen. **a** (*left ordinate)*, calcein-labeled Mac-1-HEK293 cells were preincubated with different concentrations of dynorphin A (black bar), dynorphin B (open bar) or control peptide (gray bar) for 10 min at 22 °C and added to the wells of microtiter plates coated with 2.5 μg/mL fibrinogen. Cells were allowed to adhere for 30 minutes at 37 °C before removing nonadherent cells. Data are expressed as a percentage of control adhesion (in the absence of peptides) and are the mean ± S.E. of three individual experiments performed with triplicate determinations in each experiment. **p < 0.01; ns, no significant difference. **b** (*right ordinate)*, Mac-1-expressing HEK293 cells were preincubated with dynorphin A and B as described in *A*, centrifuged for 5 min at 500 × g and fluorescence of supernatants was determined. Data are expressed as a percentage of total cell fluorescence

The ability to inhibit cell adhesion is a characteristic of peptide ligands of Mac-1 [22, 23]. Consequently, we tested the effect of soluble Dyn A and Dyn B on adhesion of Mac-1-HEK293 cells to fibrinogen, the representative Mac-1 ligand [18, 20]. Dynorphins inhibited adhesion to immobilized fibrinogen in a concentration-dependent manner (Fig. 4) with Dyn A exhibiting a somewhat higher activity than Dyn B (67 ± 2 % and 56 ± 11 % at 50 μM for Dyn A and B, respectively). Control peptide DIDPKLKWD was only slightly inhibitory.

Previous investigations reported that Dyn A, but not Dyn B, enters the cells [24]. Therefore, we first examined whether these peptides at the concentrations used in adhesion and inhibition experiments described above can affect the membrane integrity causing the leakage of calcein from cells. We reasoned that if this occurred, it would result in decreased fluorescence of adherent cells giving a false impression of reduced adhesion. As shown in Fig. 4, no leakage of calcein was detected using both dynorphins, indicating that soluble peptides inhibited adhesion by blocking Mac-1.

**Fig. 5:**
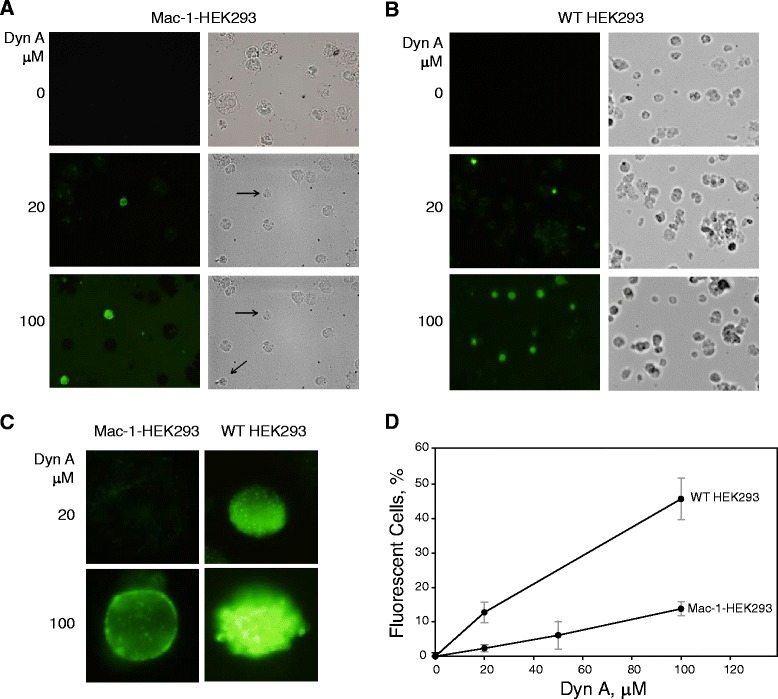
Translocation of Dyn A into cells. Mac-1-expressing (**a**) and wild-type HEK293 (**b**) cells were incubated with 20 μM and 100 μM of Dyn A for 40 min at 37 °C, fixed, permeabilized and incubated with anti-dynorphin antibodies followed by Alexa Fluor 488-conjugated secondary goat anti-rabbit antibodies. No labeling was detected in the absence of primary antibody. **c**, Enlarged images of Dyn A-accumulated Mac-1- and wild-type HEK cells shown in **a** and **b. d**, Quantification of fluorescent cells shown in **a** and **b**

To investigate whether Dyn A translocates into cells, we incubated Mac-1-HEK293 and wild-type HEK293 cells with increasing concentrations of Dyn A for 40 min at 37 °C, which corresponded to the total time the cells were exposed to Dyn A in our cell adhesion assays. Cells were then fixed and labeled with anti-dynorphin antibodies. As shown in Fig. 5a-d, a significantly smaller number of Mac-1-HEK293 cells accumulated Dyn A compared to wild-type HEK293 cells. Moreover, at 20 μM, Dyn A was primarily associated with the cell periphery of the Mac-1-HEK293 cells while entering inside wild-type HEK293 cells (Fig. 5c). This finding suggests that the presence of Mac-1 on the cell surface may retard translocation of Dyn A across the membrane, most likely by Dyn A binding to Mac-1.

### Migration of Mac-1-expressing cells to Dyn A

**Fig. 6:**
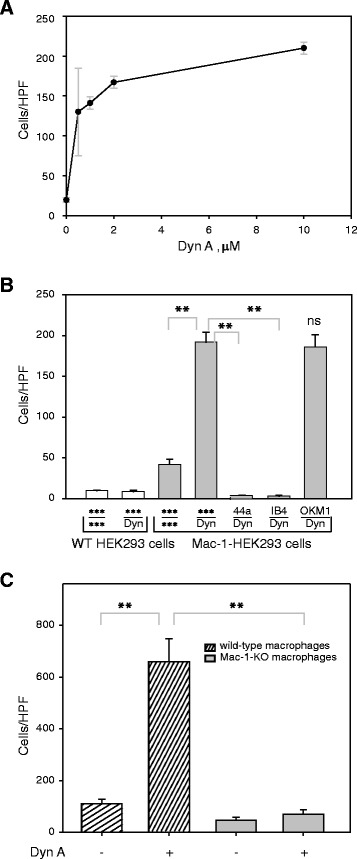
Migration of Mac-1-expressing cells to Dyn A in a Transwell system. **a**, Mac-1-expressing HEK 293 cells (100 μl at 3 × 10^6^ cell/ml) were added to the upper wells of the transwell chamber and their ability to migrate to different concentrations of Dyn A placed in the lower chamber was analyzed. After 16 hours at 37 °C, migrated cells were fixed, stained, and counted. Data are presented as numbers of migrated cells per field ± S.E. for five random fields per well. The figure is representative of 5 experiments. **b**, Mac-1-expressing or wild-type HEK 293 cells were added to the upper wells of the transwell chamber and their ability to migrate to 10 μM Dyn A placed in the lower chamber was analyzed as in *A*. Some Mac-1-HEK293 cells were pretreated with 20 μg/ml of mAbs 44a (anti-α_M_), IB4 (anti-β_2_) or OKM1 (non function-blocking anti-α_M_) or medium alone before addition to the upper wells. ***, medium alone. Data are presented as the number of migrated cells per field ± S.E. for five random fields/well from 3 separate experiments. **, p < 0.01. **c**, migration of TG-elicited macrophages isolated from the peritoneum of wild-type and Mac-1-deficient mice. Macrophages (3x10^5^) were placed in the upper chamber and allowed to migrate to Dyn A (10 μM) in the lower chamber for 90 min. The number of migrated cells was determined as in *A*. Data are presented as number of migrated cells per field ± S.E. for five random fields per well. The figure is representative of 3 experiments

Previous studies demonstrated that Dyn A (residues 1–13) was capable of inducing cell migration [25, 26]. To investigate the relationship between Mac-1, Dyn A and cell migration, we compared the ability of Mac-1-HEK293 and wild-type HEK293 cells to migrate towards Dyn A in a transwell system. These cell lines were reported to be a useful system for assessing the role of Mac-1 in migration [27, 28]. The cells were placed in the upper chambers with different concentrations of Dyn A in the lower chambers, and cells were allowed to migrate for 16 h at 37 °C. As shown in Fig. 6a, Dyn A induced a strong concentration-dependent migratory response. The concentrations as low as 0.5-1 μM induced migration of Mac-1-HEK293 cells with the response reaching saturation at 5 μM. Wild-type HEK293 cells did not migrate to Dyn A and Mac-1-HEK293 cells migrated only slightly to the medium without peptide (Fig. 6b). Direct evidence that migration of Mac-1-HEK293 cells was dependent on Mac-1 was obtained in the experiments in which anti-Mac-1 function blocking mAbs were tested (Fig. 6b). The mAbs 44a and IB4 against the α_M_ and β_2_ integrin subunits, respectively, eliminated cell migration, while non-blocking anti-α_M_ mAb OKM1 had no effect.

In another set of experiments using the transwell migration system, we evaluated migration of macrophages isolated from the peritoneum of wild-type and Mac-1-deficient mice. Purified macrophages were allowed to migrate through the 5-μm pore filter for 90 min at 37 °C. As shown in Fig. 6c, whereas wild-type microphages migrated to Dyn A, migration of Mac-1-deficient cells was significantly impaired.

**Fig. 7:**
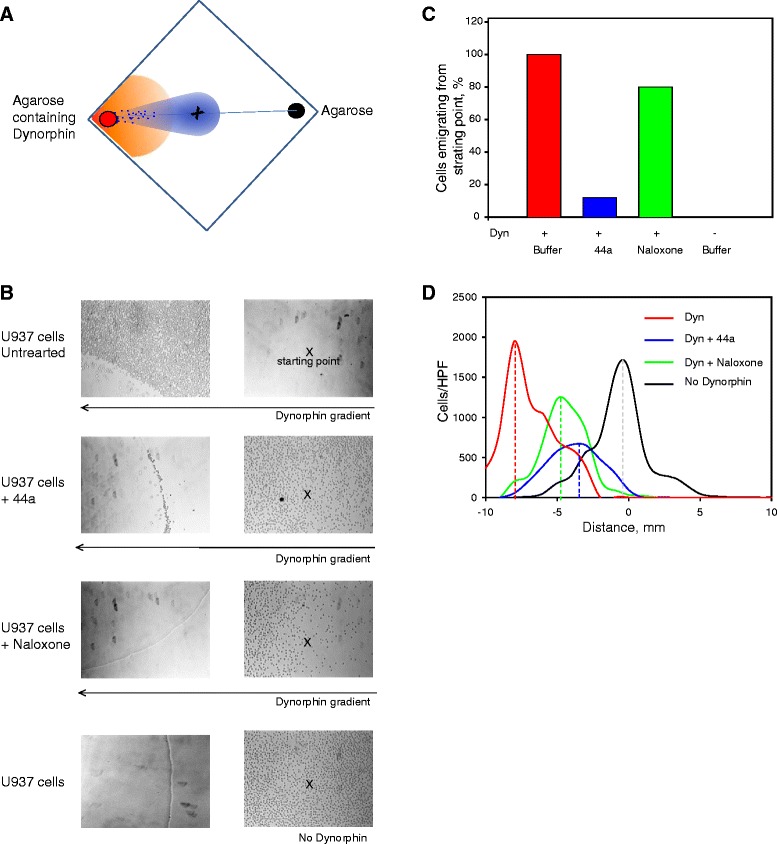
Chemotactic response of U937 cells to Dyn A. **a**, schematic representation of the experimental format. U937 cells (5x10^4^) were placed in the center of the coverslip between two agar gels, one containing Dyn A and control gel without Dyn A. After 2 h at 37 °C, a series of photographs was taken in the direction of migration at 1-mm intervals to count the number of migrating cells. **b** *,* representative images after 2 h of migration show the cells within the starting point (marked with a cross; *right panels*) and at the border of the Dyn A-containing agar drop (*left panels*). Some cells were incubated with mAb 44a (20 μg/ml) or naloxone (10 μM) for 30 min before the initiation of chemotaxis assays. Two bottom panels show the experimental condition with no Dyn A gradient. **c**, quantification of U937 cells migrated from the initial point after 2 h based on the results shown in *A* (*right panel*). Migration of control untreated U937 cells that completely migrated from the initial point of cell placement was assigned a value of 100 %. In contrast, there was no cell migration without a Dyn A gradient (0 %). Migration of cells preincubated with mAb 44a and naloxone was blocked by ~80 % and ~20 %, respectively. A representative experiment is shown. **d**, distribution of U937 cells between two gels: Dyn A-containing (*on the left*) and without Dyn A (*on the right*). “0” on *abscissa* corresponds to the initial point where cells were loaded and the numbers show the distance that cells migrated in both directions from the starting point. All cells in each of 10 consecutive fields towards the Dyn A-containing and empty gels were counted and plotted as a distance from the starting point

The ability of Dyn A to support Mac-1-mediated cell migration was further confirmed using a chemotaxis migration system in which cells migrate to agar gels impregnated with a chemotactic agent (Fig. 7a). In these experiments, Mac-1-expressing U937 monocytic cells efficiently migrated along the gradient of Dyn A with many cells arriving at the edge of the gel (Fig. 7b, *upper panel*). In contrast, no directional migration was observed when Dyn A was absent (Fig. 7b, *bottom panel*); cells remained stationary and were evenly distributed at their initial position on the coverslip. Preincubation of cells with mAb 44a resulted in inhibition of their migration compared to nontreated control cells with only few cells arriving to the Dyn A-containing drop (Fig. 7b). As shown in Fig. 7c, only ~ 15 % of cells moved from the initial field in the presence of mAb 44a, implicating Mac-1 in mediating the chemotactic response of U937 cells to Dyn A.

Leukocytes, including U937 monocytic cells, are known to express G-protein coupled κ-opioid receptors for Dyn A [8]. Therefore, we also examined the effect of naloxone, the inhibitor of these receptors. In control experiments, we have found that naloxone does not affect Mac-1 mediated cell adhesion suggesting that it does not interact with the ligand-binding site of the α_M_I-domain (not shown). Added at 10 μM, naloxone inhibited emigration of cells from the starting point, but the effect was less potent than mAb 44a, with ~20 % remaining at the start (Fig. 7b and c). However, although naloxone was a less potent inhibitor it still affected migration down the migratory path since only a few cells arrived to the Dyn A-containing drop. Therefore, we have analyzed the distribution of cells treated with either 44a or naloxone along the chemotactic gradient by determining the number of cells in each of 10 consecutive 1-mm fields. These calculations produced a Gaussian distribution of cells as a function of the distance from the starting point (Fig. 7d). The analyses demonstrated that both 44a and naloxone inhibited migration: however, while the mean distance travelled by naloxone-treated cells was ~5 mm, the mAb 44a-treated cells migrated only ~3.5 mm. The majority of untreated cells traveled ~8-mm towards the Dyn A-containing drop. Together, these results indicate that Dyn A can trigger both Mac-1 and κ-opiod receptor responses.

### Dyn A augments phagocytosis of plastic beads via Mac-1

**Fig. 8:**
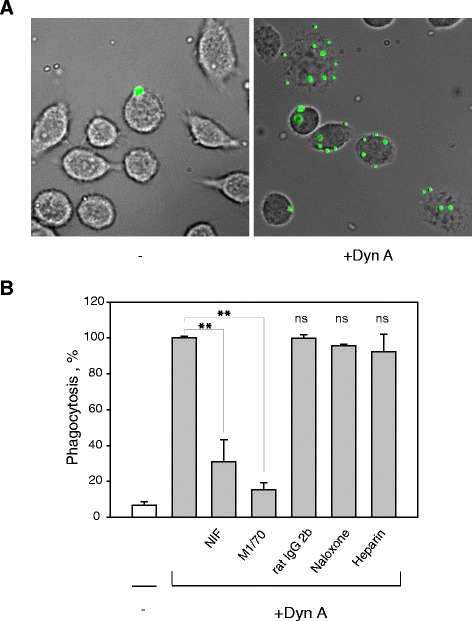
Effect of Dyn A on phagocytosis of latex beads. **a**, Fluorescent latex beads (1x10^9^/ml) were preincubated with Dyn A (50 μg/ml) for 40 min at 37 °C. Soluble peptide was removed from the beads by high-speed centrifugation. Peptide-coated beads were incubated with adherent IC-21 macrophages for 30 min at 37 °C. Nonphagocytosed beads were removed from cells by washing with PBS. IC-21 cells exposed to control (*left*) and Dyn A-treated (*right*) beads are shown. **b** *,* Peptide-coated beads were incubated with IC-21 macrophages in the presence of anti-α_M_ mAb M1/70 (20 μg/ml), heparin (20 μg/ml), NIF (2 μg/ml) or naloxone (10 μM). Rat IgG2b was used as an isotype control for anti-α_M_ mAb M1/70. Phagocytosis was quantified for five random fields per well using a 20x objective. Phagocytosis of Dyn A-treated beads was assigned as 100 %. Data shown are mean ± S.E. from three separate experiments. **p < 0.01; ns, no significant difference

We hypothesized that Dyn A immobilized on the surface of plastic beads may bind to Mac-1 on macrophages followed by their phagocytosis. To examine this possibility, control and Dyn A-treated fluorescent 1 μm beads were incubated with adherent IC-21 murine macrophages at a ratio of 20:1 for 30 min (a saturation time determined in preliminary experiments) and their phagocytosis was determined (Fig. 8a). Quantification of phagocytosed beads indicated that Dyn A augmented uptake by ~ 10-fold (Fig. 8b). To investigate whether integrin Mac-1 on macrophages is involved in promoting phagocytosis of Dyn A-coated beads, we examined the effects of mAb M1/70 (against murine α_M_) and NIF. On their own, mAb M1/70 and NIF strongly inhibited Dyn A-mediated phagocytosis by ~ 85 % and ~ 70 %, respectively (Fig. 8b). In contrast, heparin and naloxone did not decrease the number of phagocytosed beads, and an isotype control IgG2b for mAb M1/70 had no effect. Together, these results indicate that Dyn A is capable of enhancing macrophage phagocytosis via interaction with integrin Mac-1.

## Discussion

In the present study we identified opioid peptides Dyn A and Dyn B as novel ligands for leukocyte integrin Mac-1. In support of this finding, we showed that recombinant α_M_I-domain, a ligand binding region of Mac-1, interacts with Dyn A- and Dyn B. Expression of Mac-1 in HEK293 cells resulted in Mac-1-mediated cell adhesion to immobilized Dyn A and Dyn B, while related integrin LFA-1 (α_L_β_2,_ CD11a/CD18) or Mac-1 in which the α_M_I-domain was deleted by genetic manipulation, supported a significantly lower level of adhesion to Dyn A and did not bind Dyn B. We found that Dyn A supports chemotactic responses of Mac-1-expressing HEK293 cells and murine macrophages, which are entirely dependent on Mac-1. In U937 monocytic cells, the Mac-1-mediated migration was partially inhibited by naloxone, suggesting the involvement of both Mac-1 and opioid receptors. In addition, Dyn A enhanced phagocytosis of latex beads by murine macrophages in a Mac-1 dependent and naloxone-independent manner. These observations establish Mac-1 as a receptor for Dyn A and Dyn B and suggest a role for the Mac-1-Dyn A interactions in the induction of nonopioid receptor-dependent effects in leukocytes.

The characteristic feature of Mac-1 ligands is the presence of basic residues surrounded by hydrophobic residues which form short α_M_I-domain recognition motifs [17, 18]. The sequences enriched in such motifs are abundant in many cationic proteins and peptides that are stored in neutrophil granules, including elastase, myeloperoxidase, cathepsin G and others. Inspection of the Dyn AB (residues 1–32) sequence revealed that it contains several typical α_M_I-domain recognition motifs in both the N-terminal and C-terminal parts, including ^4^FLRRIRPKLK^13^ and ^23^FLRRQFKVV^31^, suggesting that Dyn A and Dyn B, encompassing residues 1–17 and 20–32, respectively, may be Mac-1 ligands. Indeed, screening of the peptide libraries spanning the sequence of Dyn AB showed that recombinant α_M_I-domain interacted with many overlapping peptides, with somewhat higher affinity for those spanning the sequences of Dyn A and Dyn B (Fig. 1b). The α_M_I-domain binding to dynorphin-derived peptides was specific inasmuch as mutations of positively charged residues, the critical determinants of the α_M_I-domain recognition specificity, strongly reduced the interaction. The ability of Dyn A and Dyn B to interact with the whole receptor was shown in adhesion assays in which Dyn A and Dyn B supported efficient adhesion of Mac-1 expressing cells. The specificity of Mac-1 interaction with Dyn A and Dyn B has been further confirmed by blocking the interaction with anti-Mac-1 reagents. Consistent with our previous results in other Mac-1-ligand systems [21, 22], soluble heparin potentiated the inhibitory effect of function-blocking anti-Mac-1 mAb suggesting that cell surface HSPGs may cooperate with Mac-1 in recognition of positively charged dynorphins. The behavior of Dyn A and Dyn B in the experiments with isolated α_M_I-domain and whole receptors expressed on cells recapitulates that of other well-characterized Mac-1 peptide ligands, including the fibrinogen peptide γ^383^TKIIPFNRLTIG^395^ (P2-C) [23, 18] and the growth factor CCN1-derived peptide SSVKKYRPKYCGS (CCN1-H2) [22], both of which contain typical α_M_I-domain recognition motifs.

Numerous studies reported that Dyn A has immunomodulatory properties and elicits various responses in cultured cells and isolated murine macrophages, including migration, phagocytosis, oxidative burst, degranulation and expression of cytokines [5, 7, 9, 14, 15]. Dyn A binds preferentially to the k-opioid receptors that have been identified on resting mouse macrophages, macrophage cell lines and neutrophils (Reviewed in [29–31]). In addition to the k-opioid receptors, for which Dyn A shows higher affinity, it can bind to μ- and δ-opioid receptors [32] that have also been detected on monocytes/macrophages from several species [30]. However, many responses induced by Dyn A in myeloid cells are naloxone-insensitive [7, 11, 15] suggesting that it can interact with other receptors. Macrophages and neutrophils abundantly express integrin Mac-1 (CD11b/CD18), which is a common marker of these cells. It is well known that ligand engagement by Mac-1 initiates intracellular signaling that regulates many leukocyte responses [16, 33]. A striking similarity between responses induced in macrophages and neutrophils by Dyn A and by many Mac-1 ligands suggests that binding of Dyn A to Mac-1 may mediate non-opioid reactions. Furthermore, Dyn A induces degranulation of mast cells, which also express Mac-1 [34], suggesting that in these cells Dyn A may act through Mac-1.

Consistent with the idea that Dyn A may mediate responses via Mac-1, migration of Mac-1-expressing HEK293 cells was completely blocked by anti-Mac-1 function-blocking antibodies (Figs. 6 and 7). HEK293 cells transfected with Mac-1 are often used in structural and functional analyses of this integrin to avoid the confounding effects of other leukocyte receptors. An additional advantage of these cells is that they do not express opioid or NMDA receptors [35] which are also a target for dynorphins (reviewed in [1]) and which otherwise may be present on cultured macrophage-like cell lines or monocytes/macrophages isolated from blood. Nevertheless, we found that the effect of Dyn A on migration of thioglycollate-elicited mouse peritoneal macrophages was solely dependent on Mac-1, inasmuch as wild-type, but not Mac-1-deficient macrophages, migrated to Dyn A. In contrast, migration of human U937 monocytic cells seems to depend on both opioid receptors and Mac-1. Previous studies that assessed expression of opioid receptors on human peripheral blood monocytes produced conflicting results. Williams et al. [36] reported that peripheral blood mononuclear cells (PBMC) do not display classical opioid receptors, while another group showed the presence of transcripts for μ-opiod receptors not only in lymphocytes, which constitute the majority of PBMC, but also in isolated monocytes and neutrophils [37]. It was proposed that even though resting blood monocytes do not express opioid receptors, their expression may increase after proinflammatory stimulation [36]. Furthermore, cultured U937 monocytic cells may differ from normal cells with regard to expression of opioid receptors. Although it is clear that Dyn A binds Mac-1 and induces migration of Mac-1 expressing cells, the relationship between opioid receptors and Mac-1 on human peripheral blood monocytes/macrophages remains to be more clearly defined.

Previous studies demonstrated that Dyn A is able to translocate across the plasma membrane in a number of cells [24]. In agreement with this observation, we showed that Dyn A penetrates wild-type HEK293 cells. However, we also consistently observed that the penetration of Dyn A was strongly reduced in the Mac-1-expressing HEK293 cells. Moreover, in these cells, Dyn A was detected as a rim around the cell periphery suggesting that binding of Dyn A to Mac-1 may delay translocation of the peptide. The potential of dynorphins to penetrate into cells has been proposed to correlate with their ability to induce non-opioid effects in animals [24]. However, the fact that Dyn A-induced migration of Mac-1-expressing HEK293 cells was fully blocked by anti-Mac-1 function-blocking mAbs suggests that, at least in these cells, the response was mediated by binding of Dyn A to Mac-1. Nevertheless, although the observed pattern of Dyn A distribution at the plasma membrane of Mac-1-expressing HEK293 cells may potentially serve as evidence for the interaction of Dyn A with Mac-1 additional studies may help to define the role of Mac-1 in translocation of Dyn A in Mac-1-expressing blood leukocytes.

A distinctive property of Dyn A, as well as other opioid peptides, is that they are stored in neutrophil granules and in monocyte/macrophages, and released from these cells upon stimulation by various proinflammatory agonists (reviewed in [4, 38]). Dynorphins were also found in mouse monocyte/macrophages recruited to the site of inflammation [39, 40]. Many cationic proteins stored in primary (elastase, myeloperoxidase, cathepsin G and azurocidin) and specific (hCAP18/LL-37) neutrophil granules are Mac-1 ligands [17, 41, 42] enriched in Mac-1 recognition sequences similar to those found in Dyn A [17]. Furthermore, like Dyn A, they induce immunomodulatory responses in monocyte/macrophages [43–45]. It is well known that the neutrophil influx from the circulation represents the first wave of leukocyte migration to the site of injury followed by a second wave of monocyte extravasation. It has been proposed that neutrophils trigger this cellular switch by releasing granule products and, thus, pave the way for migration of monocyte/macrophages [46, 47]. It is tempting to speculate that dynorphins, similar to other cationic peptide/proteins, fulfill a common function of alarming the immune system by evoking leukocyte responses via Mac-1. However, further studies in dynorphin knock out mice are required to investigate whether the interaction of dynorphins with Mac-1 is biologically relevant.

One important property of Dyn A that is not shared by other cationic peptide/proteins is that it is a bi-functional molecule, i.e., it acts directly on opioid receptors and also displays other activities. Similar to other cationic peptides [48, 49], Dyn A can exert some effects essential for host defense. Dyn A, and to less extent Dyn B, have been shown to enhance phagocytosis by mouse peritoneal macrophages [7]. Interestingly, the phagocytic activity strongly correlated with the presence of the ^6^ArgArgIle^8^ sequence in Dyn A, which represents a core structure of the α_M_I-domain recognition motif. Indeed, we have shown that Dyn A strongly enhanced phagocytosis of latex beads by binding to Mac-1 (Fig. 8), a well-known phagocytic receptor [50, 51]. Dyn A and Dyn B seems to be unique among opioid peptide family members since in contrast to β-endorphin and Met-enkephalin, they contain readily identifiable sequences typical for the α_M_I-domain recognition motifs. Nevertheless, the presence of short sequences containing lysine and adjacent hydrophobic residues (LeuPheLys and IleIleLys) in β-endorphin and arginine surrounded by hydrophobic residues (PheMetArgPhe) in Met-enkephalin-ArgPhe is intriguing and may warrant the investigation of their Mac-1 binding potential.

Although neutrophils and macrophages contain Dyn A which may contribute to eliciting leukocyte responses in inflamed peripheral tissues, the major source of this peptide is the CNS (reviewed in [1]). The concentration of Dyn A within dense core vesicles of neurons was estimated to be 1 mM [52] and the levels of the prodynorphin precursor within the vesicles appears to be even higher. It has been proposed that the concentration of Dyn A within the extracellular space of the CNS is greater than 10 μM and could significantly increase following spinal cord or traumatic brain injuries. With such high levels, it is possible that Dyn A released during these pathologies may not only perform its primary role in pain reduction, but also act as a chemotactic signal attracting neutrophils and monocytes to sites of injury. Indeed, spinal cord injury is known to induce a strong inflammatory response with invasion of neutrophils and monocytes known as secondary injury [53]. Further studies utilizing dynorphin, KOR and Mac-1 knockout mice are likely to be a useful strategy to investigate the role of dynorphins using the experimental models of spinal cord injury and other models of inflammation.

The concept that the immune system can communicate with peripheral sensory neurons to modulate pain has emerged from numerous studies that explored the role of opioid-containing neutrophils in early inflammation and monocytes/macrophages at later stages in inducing antinociception [54, 4]. The finding in the present study that Dyn A and Dyn B are ligands for integrin Mac-1, the major adhesion and signaling receptor expressed on myeloid cells, coupled with the data that Dyn A-Mac-1 interactions may initiate proinflammatory responses in these cells, establishes a hitherto unrecognized molecular link between opioid and immune systems, affording new insights into the function of the neuro-immune axis. Although the precise role of Dyn A in triggering Mac-1-mediated immunomodulatory reactions remains to be further defined *in vivo*, the fact that the amino acid sequence of Dyn A and Dyn B is highly conserved evolutionarily [55] may point to a special role of these multifunctional peptides in the complex network of opioid peptides and their receptors on immune cells.

## Conclusions

The results from these studies identify opioid peptides dynorphin A and B as novel ligands for leukocyte integrin Mac-1 (α_M_β_2_, CD11b/CD18). We have found that both dynorphin A and B contain typical recognition motifs for the α_M_I-domain, the ligand binding region of Mac-1, and demonstrated that both opioid peptides directly interact with recombinant α_M_I-domain and whole receptors expressed on cells. Focusing further on dynorphin A, we have shown that at variance with other Mac-1 ligands, dynorphin A does not require the active state of the α_M_I-domain. In functional assays, dynorphin A can support a Mac-1-mediated chemotactic cell migration and enhance phagocytosis by murine macrophages by binding to Mac-1. These findings suggest a role for the Mac-1-Dyn A interactions in the induction of nonopioid receptor-dependent effects in leukocytes.

## Methods

### Peptides, proteins and monoclonal antibodies

Dyn A (YGGFLRRIRPKLKWDNQ), Dyn B (YGGFLRRQFKVVT), and control peptide (DIDPKLKWD) were purchased from AnaSpec, Inc (San Jose, CA). Fibrinogen, depleted of fibronectin and plasminogen, was obtained from Enzyme Research Laboratories (South Bend, IN). The D_100_ (mol. weight 100,000 Da) fragment of fibrinogen was prepared by digestion of fibrinogen with plasmin [56]. The mAbs 44a and OKM1, directed against the α_M_-integrin subunit and mAb IB4, directed against the β_2_-integrin subunit, were purified from the conditioned media of the hybridoma cell lines obtained from ATCC (Manassas, VA) using protein G agarose (GenScript, Piscataway, NJ). Polyclonal anti-Dyn A antibody (ab 82509) was from Abcam (Cambridge, MA). Neutrophil inhibitory factor (NIF) was a gift from Corvas International. The anti-GST mAb was purchased from Pierce Precision Antibody (Rockford, IL). BSA, polyvinylpyrrolidone (PVP), fMLP, heparin, and chondroitin sulfate A and B were obtained from Sigma (St. Louis, MO). Calcein AM was purchased from Molecular Probes (Life Technologies, Grand Island, NY). Recombinant α_M_I-domains in the active (residues Glu^123^-Lys^315^) and non-active conformation (residues Gln^119^-Glu^333^) were expressed and purified from *E. coli* lysates as fusion proteins with glutathione S-transferase (GST) using affinity chromatography as previously described [57].

### Synthesis of cellulose-bound peptide libraries

Peptide libraries derived from dynorphin AB assembled on cellulose membrane supports were prepared by parallel spot synthesis as previously described [58, 18]. Peptides were COOH-terminally attached to cellulose via a (β-Ala)_2_ spacer and were acetylated N-terminally. The membrane-bound peptides were tested for their ability to bind the α_M_I-domain according to a previously described procedure [18]. In brief, the membrane was blocked with 1 % BSA and then incubated with 10 μg/ml of ^125^I-labeled α_M_I-domain in TBS containing 1 mM MgCl_2_. After washing, the membrane was dried and the α_M_I-domain binding was visualized by autoradiography.

### Solid-phase binding assays

Microtiter plates were coated with different concentrations of the fibrinogen D_100_ fragment or Dyn A overnight at 4 °C and post-coated with 3 % BSA for 1 h. The GST-α_M_I-domains in 20 mM Tris–HCl, pH 7.4, 100 mM NaCl, 1 mM MgCl_2_, 1 mM CaCl_2_, 0.05 % Tween 20, and 5 % glycerol were added to the wells and incubated for 1.5 h at 22 °C. After washing, the plates were incubated with an anti-GST mAb at a 1:5000 dilution for 1 h at 22 °C. After washing, goat anti-mouse IgG conjugated to alkaline phosphatase was added for 1 h and the binding of the α_M_I-domains was detected by reaction with p-nitrophenyl phosphate measuring the absorbance at 405 nm. Background binding to BSA was subtracted.

### Cells

Human embryonic kidney cells (HEK293), Mac-1*-*expressing HEK293 cells (Mac-1-HEK293), LFA-1 (CD11a/CD18) and HEK293 cells expressing the α_M_ subunit lacking the α_M_I-domain [20] were maintained in DMEM (Mediatech Inc*,* Manassas, VA) supplemented with 10 % fetal bovine serum and antibiotics. U937 monocytic cells were grown in RPMI containing 10 % fetal bovine serum and antibiotics. Neutrophils were isolated under sterile conditions from human peripheral blood obtained from consenting volunteers as described [59]. Peritoneal macrophages were isolated from wild-type and Mac-1-deficient mice (The Jackson Laboratories) 3 days after the injection of 4 % thioglycollate (TG). Macrophages were separated from lymphocytes using a mouse PE selection kit (STEMCELL Technologies, Vancouver, BC, Canada).

### Cell adhesion

Adhesion assays were performed essentially as described previously [28, 20]. Briefly, the wells of 96-well polysterene microtiter plates (Immulon 4HBX, Thermo) were coated with various concentrations of Dyn A peptide, Dyn B, control peptide, or D_100_ fibrinogen fragment for 3 h at 37 °C, and then post-coated with 1 % PVP for 1 h at 37 °C. Cells were labeled with 10 μM calcein for 30 min at 37 °C and washed twice with Hanks’ Balanced salt solution (HBSS) containing 0.1 % BSA. Aliquots (100 μl) of labeled cells (5x10^5^/ml) were added to each well and allowed to adhere for 30 min at 37 °C. The non-adherent cells were removed by two washes with PBS. Fluorescence was measured in a CytoFluorII fluorescence plate reader (Perceptive Biosystems, Framingham, MA). In inhibition experiments, cells were mixed with different concentrations of peptides, NIF or mAbs for 20 min at 22 °C before they were added to the wells coated with adhesive substrates. In a separate set of experiments, after coating plates with dynorphins and post-coating with 1 % PVP, plates were incubated with 100 μl of 10 μg/ml chondroitin sulfate A, chondroitin sulfate B, or heparin for 20 min at 22 °C. After aspirating the solution, labeled cells were added and cell adhesion was analyzed as above.

### Phagocytosis assays

A suspension of fluorescent 1.0 μM latex beads (1x10^9^/ml) (FluoSpheres sulfate microspheres, Life Technologies, Carlsbad, CA) was incubated with 50 μg/ml of Dyn A for 40 min at 37 °C, then unbound peptide was removed by centrifugation. IC-21 murine macrophages (5 × 10^5^/ml) were adhered on the cover glass for 30 min and 3 × 10^6^ of peptide-coated beads were added to adherent IC-21 cells. After incubating for 30 minutes at 37 °C, nonphagocytosed beads were separated from macrophages by washing with 3x1 ml PBS and phagocytosed beads were counted in the presence of trypan blue. In inhibition experiments, cells were mixed with mAbs, NIF, heparin or naloxone for 15 min at 22 °C before addition of the fluorescent beads. The ratio of beads per macrophage was quantified by taking photographs of five fields for each well using a Leica DM4000 B microscope (Leica Microsystems, Buffalo Grove, IL) with a 20 × objective.

### Transwell migration assays

Migration assays with wild-type and Mac-1-expressing HEK293 cells and purified macrophages using Transwell inserts (5 μm pore size) were performed as previously described [27, 28]. Briefly, cells (3 × 10^5^) were placed in the upper chamber of the Transwell system and lower chambers contained 600 μl of DMEM medium + 10 μM Dyn A. For inhibition experiments, cells were pretreated with 20 μg/ml of each function blocking anti-α_M_ mAb 44a, anti-β_2_ mAb IB4 or noninhibitory mAb OKM1 for 30 min before their addition to the upper chamber of the transwell system with 10 μM Dyn A in the lower chamber. Migration was allowed to proceed for 16 h and 90 min for Mac-expressing HEK293 cells and macrophages, respectively, at 37 °C. The cells in the upper wells were removed by wiping with a cotton swab and cells adherent to the underside of the filter were fixed with paraformaldehyde and stained with Hematoxylin. The photographs were taken using a 20x objective and cells in three random fields and cells were counted. Migration data are presented as mean cells/field ± SE from three experiments.

### Chemotaxis migration assay

Chemotaxis assays using U937 monocytic cells were performed on 22 × 22 mm cover slips. 1 % agarose (Life Technologies, Carlsbad, CA) was dissolved in HBSS by heating. After cooling, the agarose solution was mixed with Dyn A to obtain a final concentration of 10 μM. A 10 μl drop of warm agarose solution containing Dyn A was placed at one corner of the cover glass at ~1.5 mm from the edge. A control agarose drop was placed diagonally at the opposite corner of the cover glass. Agarose was allowed to solidify for 5 min, and the cover glasses were placed into wells of the 6-well plate filled with 5 ml of RPMI 1640 + 10 % FBS. A 10-μl aliquot of U937 cells (5x10^4^) was loaded in the center of the cover glass and the plate was incubated for 2 h at 37 °C in a humidified atmosphere containing 5 % CO_2_. In this experimental format, the cells sediment approximately 5 min after loading, forming an ~4 mm circle, and begin to migrate towards the Dyn A-containing agarose drop. In inhibition experiments, cells were incubated with 44a (20 μg/ml) or Naloxone (10 μM) for 30 min before adding them to the cover glass. The photographs were taken at 1-mm intervals in the direction of cell migration towards the dynorphin-containing or control agarose drops without dynorphin A. The number of cells at the starting point and in each of 10 consecutive fields towards the dynorphin-containing and empty gels were counted and plotted as a distance from the starting point.

### Immunolabeling of dynorphin A translocated into cells

Mac-1-HEK 293 and wild-type HEK 293 cells were incubated for 10 min with different concentrations (0–100 μM) of Dyn A and allowed to adhere to glass coverslips coated with poly-Lysine for 30 min. Adherent cells were fixed in 2 % paraformaldehyde solution, permeabilized with 0.1 % Tween-20, and blocked with 10 % normal goat serum plus 1 % BSA. The coverslips were incubated with 5 μg/ml polyclonal anti-Dyn A antibody followed by an secondary goat anti-rabbit antibody conjugated to Alexa Fluor 488 (1:1000). Images were taken using a Leica DM 4000B microscope (Leica, Buffalo Grove, IL).

### Statistical analysis

All data are presented as the mean ± S.E. The statistical differences between two groups were determined using a Student’s *t*-test from SigmaPlot 11.0 software (Systat Software, San Jose, CA). For multiple comparisons, the Bonferroni correction method was used. Differences were considered significant if p-value was less than 0.05.

## Additional file

Additional file 1: Figure S1. Identification of residues in dynorphin A critical for *α*_M_I-domain binding. The dynorphin AB-based substitutional peptide libraries with single, double or triple mutations of basic residues were prepared by SPOT synthesis as described in Experimental Procedures. Mutated amino acid residues are shown in red. The membranes with synthesized peptide libraries were incubated with ^125^I-labeled *α*_M_I-domain and subjected to autoradiography. The *α*_M_I-domain binding was analyzed by densitometry and expressed in arbitrary units with the strongest binding to wild-type peptides assigning a value of 4+. The peptides that partially lost their ability to bind the *α*_M_I-domain were grouped according to their activity and marked as 3+, 2+ and +. The peptides that lost their ability to bind the *α*_M_I-domain are marked with “NB “.
