# Supplementary material for: The opioid peptide dynorphin A induces leukocyte responses via integrin Mac-1 (αMβ2, CD11b/CD18)
Source: Mol Pain. 2015 Jun 3;11:33. doi: 10.1186/s12990-015-0027-0 (PMC4481117; doi:10.1186/s12990-015-0027-0)
Supplement: Additional file 1: Figure S1. — Identification of residues in dynorphin A critical for α MI-domain binding. The dynorphin AB-based substitutional peptide libraries with single, double or triple mutations of basic residues were prepared by SPOT synthesis as described in Experimental Procedures. Mutated amino acid residues are shown in red. The membranes with synthesized peptide libraries were incubated with 125I-labeled α MI-domain and subjected to autoradiography. The α MI-domain binding was analyzed by densitometry and expressed in arbitrary units with the strongest binding to wild-type peptides assigning a value of 4+. The peptides that partially lost their ability to bind the α MI-domain were grouped according to their activity and marked as 3+, 2+ and +. The peptides that lost their ability to bind the α MI-domain are marked with “NB “. [file 12990_2015_27_MOESM1_ESM.pdf]

THE OPIOID PEPTIDE DYNORPHIN A INDUCES LEUKOCYTE RESPONSES VIA INTEGRIN Mac-1 ($\alpha_M\beta_2$, CD11b/CD18)

Nataly P. Podolnikova, Julie A. Brothwell and Tatiana P. Ugarova

	Peptide	Signal	Peptide	Signal	Peptide	Signal	Peptide	Signal	Peptide	Signal	Peptide	Signal	Peptide	Signal	Peptide	Signal	Peptide	Signal
Wild Type	YGGFLRRIR	4	FLRRIRPLK	4	RIRPKLKWD	4	PKLKWDNQK	4	KWDNQKRYG	4	NQKRYGGFL	3	RYGGFLRRQ	4	GFLRRQFKV	3	RRQFKVVGG	1
Mutants	YGGFLDRIR	1	FLDRIRPLK	3	DIRPKLKWD	3	PELKWDNQK	NB	EWDNQKRYG	3	NQERYGGFL	NB	DYGGFLRRQ	3	GFLDRQFKV	3	DRQFKVVGG	2
	YGGFLRDIR	1	FLRDIRPLK	2	RIDPKLKWD	2	PKLEWDNQK	NB	KWDNQERYG	NB	NQKDYGGFL	NB	RYGGFLDRQ	2	GFLRDQFKV	2	RDQFKVVGG	1
	YGGFLRRID	2	FLRRIDPLK	2	DIDPKLKWD	NB	PKLKWDNQE	NB	EWDNQERYG	NB	NQEDYGGFL	NB	RYGGFLRDQ	1	GFLDDQFKV	NB	DDQFKVVGG	NB
	YGGFLDDIR	NB	FLDRIRPLE	NB	DIRPELKWD	NB	PELEWDNQK	NB	EWDNQKDYG	NB			DYGGFLDRQ	NB	GFLDRQFEV	NB	DRQFEVVGG	NB
	YGGFLRDID	NB	FLRDIRPLE	NB	RIDPELKWD	NB	PKLEWDNQE	NB	KWDNQEDYG	NB			RYGGFLDDQ	NB	GFLRDQFEV	NB	RDQFEVVGG	NB
	YGGFLDRID	NB	FLRRIDPLE	NB	DIDPELKWD	NB	PELKWDNQE	NB	EWDNQEDYG	NB			DYGGFLRDQ	NB	GFLDDQFEV	NB	DDQFEVVGG	NB
	YGGFLDDID	NB	FLDDIRPLK	1	DIRPKLEWD	NB	PELEWDNQE	NB	KWDNQKDYG	NB			DYGGFLDDQ	NB	GFLRRQFEV	1	RRQFEVVGG	1
			FLDRIDPLK	1	RIDPKLEWD	NB												
			FLRDIDPLK	1	DIDPKLEWD	NB												
			FLDDIRPLE	NB	RIRPELEWD	NB												
			FLDRIDPLE	NB	DIDPELEWD	NB												
			FLRDIDPLE	NB	DIRPELEWD	NB												
			FLDDIDPLK	NB	RIDPELEWD	NB												
			FLDDIDPLE	NB	RIRPKLEWD	NB												
			FLRRIRPLE	2	RIRPELKWD	1												

Figure S1. Identification of residues in dynorphin A critical for αM I-domain binding. The dynorphin AB-based substitutional peptide libraries with single, double or triple mutations of basic residues were prepared by SPOT synthesis as described in Experimental Procedures. Mutated amino acid residues are shown in red. The membranes with synthesized peptide libraries were incubated with ^{125}I-labeled α_MI domain and subjected to autoradiography. The α_MI-domain binding was analyzed by densitometry and expressed in arbitrary units with the strongest binding to wild-type peptides assigning a value of 4+. The peptides that partially lost their ability to bind the α_MI-domain were grouped according to their activity and marked as 3+, 2+ and +. The peptides that lost their ability to bind the α_MI-domain are marked with “NB “.
